# Advances in the localization of pulmonary nodules: a comprehensive review

**DOI:** 10.1186/s13019-024-02911-8

**Published:** 2024-06-27

**Authors:** Yafang Wang, Enguo Chen

**Affiliations:** Department of Respiratory and Critical Care Medicine, Sir Run Run Shaw Hospital, Medical School of Zhejiang University, Shangcheng District, No. 3 Qingchun East Road, Hangzhou, 310000 China

**Keywords:** CT, Pulmonary nodule localization, VATS

## Abstract

In recent years, with the widespread use of chest CT, the detection rate of pulmonary nodules has significantly increased (Abtin and Brown, J Clin Oncol 31:1002-8, 2013). Video-assisted thoracoscopic surgery (VATS) is the most commonly used method for suspected malignant nodules. However, for nodules with a diameter less than 1 cm, or located more than 1.5 cm from the pleural edge, especially ground-glass nodules, it is challenging to achieve precise intraoperative localization by manual palpation (Ciriaco et al., Eur J Cardiothorac Surg 25:429-33, 2004). Therefore, preoperative accurate localization of such nodules becomes a necessary condition for precise resection. This article provides a comprehensive review and analysis of the research progress in pulmonary nodule localization, focusing on four major localization techniques: Percutaneous puncture-assisted localization, Bronchoscopic preoperative pulmonary nodule localization, 3D Printing-Assisted Localization, and intraoperative ultrasound-guided pulmonary nodule localization.

## Percutaneous puncture-assisted localization

The most commonly used tool for percutaneous puncture localization is CT. CT-guided percutaneous puncture localization of pulmonary nodules is the earliest and most widely used localization method. It can be classified into six categories based on the materials used for localization.

### Metallic materials

**Fig. 1:**
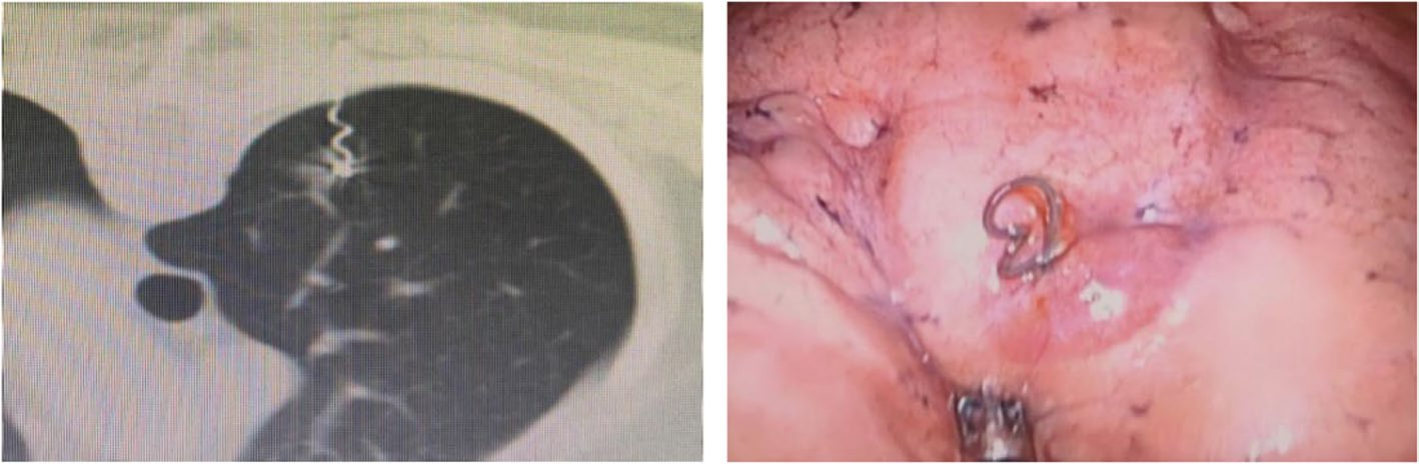
The CT-guided memory alloy coil localization [3]

Various metallic localization materials, including hook-wire, traditional micro-spring coils, four-hook localization needles, and memory spring coils, are employed. Hook-wire, the oldest and most widely used metallic material, involves leaving a segment of wire outside the chest wall after percutaneous placement, requiring fixation by bending it closely to the skin. However, hook-wire has drawbacks such as high displacement and detachment rates, noticeable pain, and a risk of air embolism [1]. The use of traditional microcoils is secondly to hook-wires. It has lower risks of displacement, pain, and bleeding compared to hook-wires. The main drawback is that if the coil is positioned too deep and cannot be found by palpation or localization puncture hole during surgery, fluoroscopy or CT confirmation of the position is required. Four-hook needles and memory shape memory coils are newly developed localization materials in recent years. Unlike traditional microcoils, they do not enter the lung entirely but leave a segment outside the visceral pleura, allowing for rapid intraoperative localization without the need for fluoroscopy or CT confirmation. The front end of the four-hook needle has metal hooks that can better anchor the pulmonary nodule and are less likely to become unhooked. The tail end is a marker thread placed on the surface of the visceral pleura, aiding precise intraoperative localization. Memory shape memory coils are made of a new type of nickel-titanium alloy material. After localization, they can form a unique dumbbell-shaped structure, and the tail structure outside the visceral pleura facilitates rapid intraoperative localization of the nodule by the surgeon. Recent developments include four-hook localization needles and memory spring coils, which have shown good safety and effectiveness, making them worthy of clinical promotion [2, 3]. Figure 1 shows the CT-guide memory alloy coil localization.

### Dyes

**Fig. 2:**
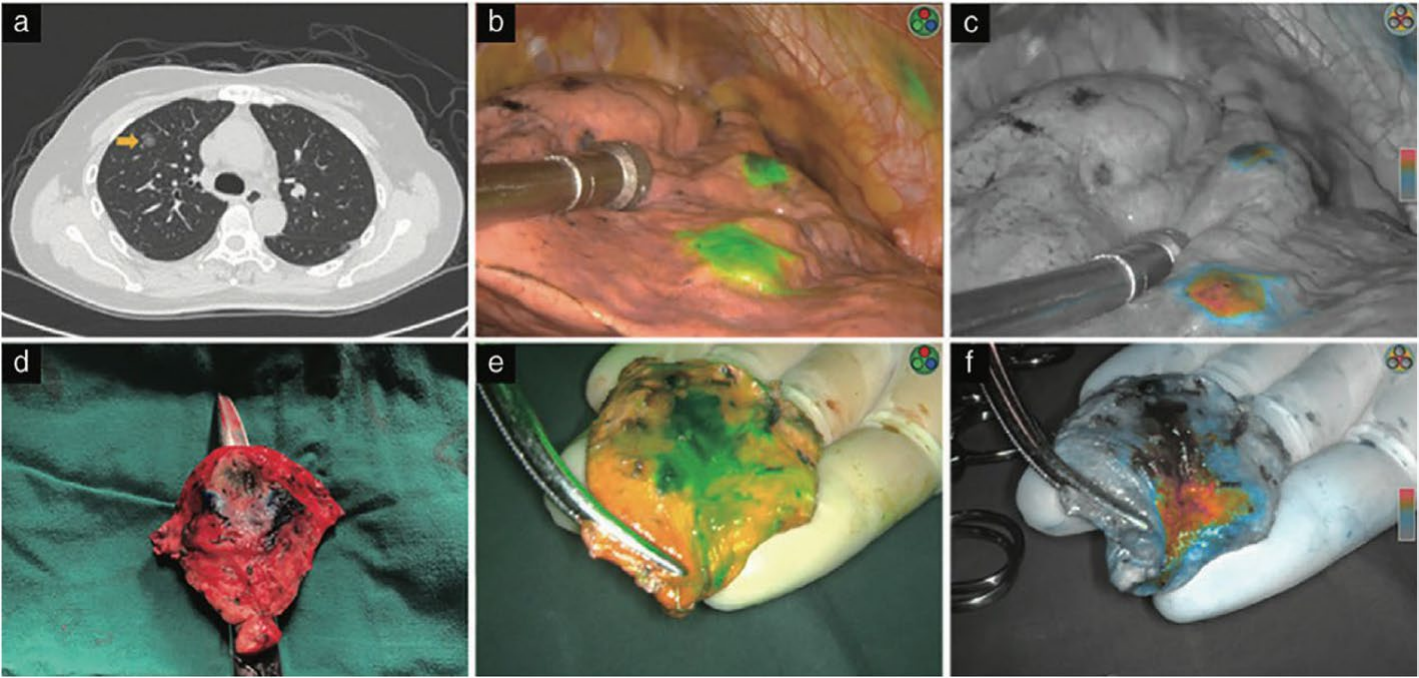
The CT-guided indocyanine green localization [5]

CT-guided percutaneous puncture injection of dyes is also widely used in clinical practice. The most commonly used dyes are methylene blue and indocyanine green (ICG). Methylene blue was used more frequently in the early stages, but its rapid diffusion after coloring requires strict surgical interval requirements. Moreover, for patients with obvious carbon particle deposition, the color recognition after methylene blue localization is low, leading to potential localization failure [4]. ICG is a tricarbocyanine dye with excellent water solubility and near-infrared absorption and emission fluorescence properties. After entering the body, ICG binds tightly to plasma proteins, has deep tissue penetration, and can be visualized under fluorescence thoracoscopy during surgery. ICG localization can be used for wedge resection and segmental resection of the lung. Preoperative CT-guided percutaneous injection of ICG is used for wedge resection, while intravenous injection of ICG during surgery is used for segmental resection. ICG fluorescence can be maintained locally for a long time and has certain advantages over methylene blue, with high localization success rate and good safety [5]. It is important to control the injection dose of ICG. Too much will cause the fluorescence to diffuse in the thoracic cavity and affect positioning, and too little will make it impossible to locate. The main requirement for ICG localization is the use of fluorescence thoracoscopy. Figure 2 shows the CT-guide indocyanine green localization.

### Medical bio-glue

**Fig. 3:**
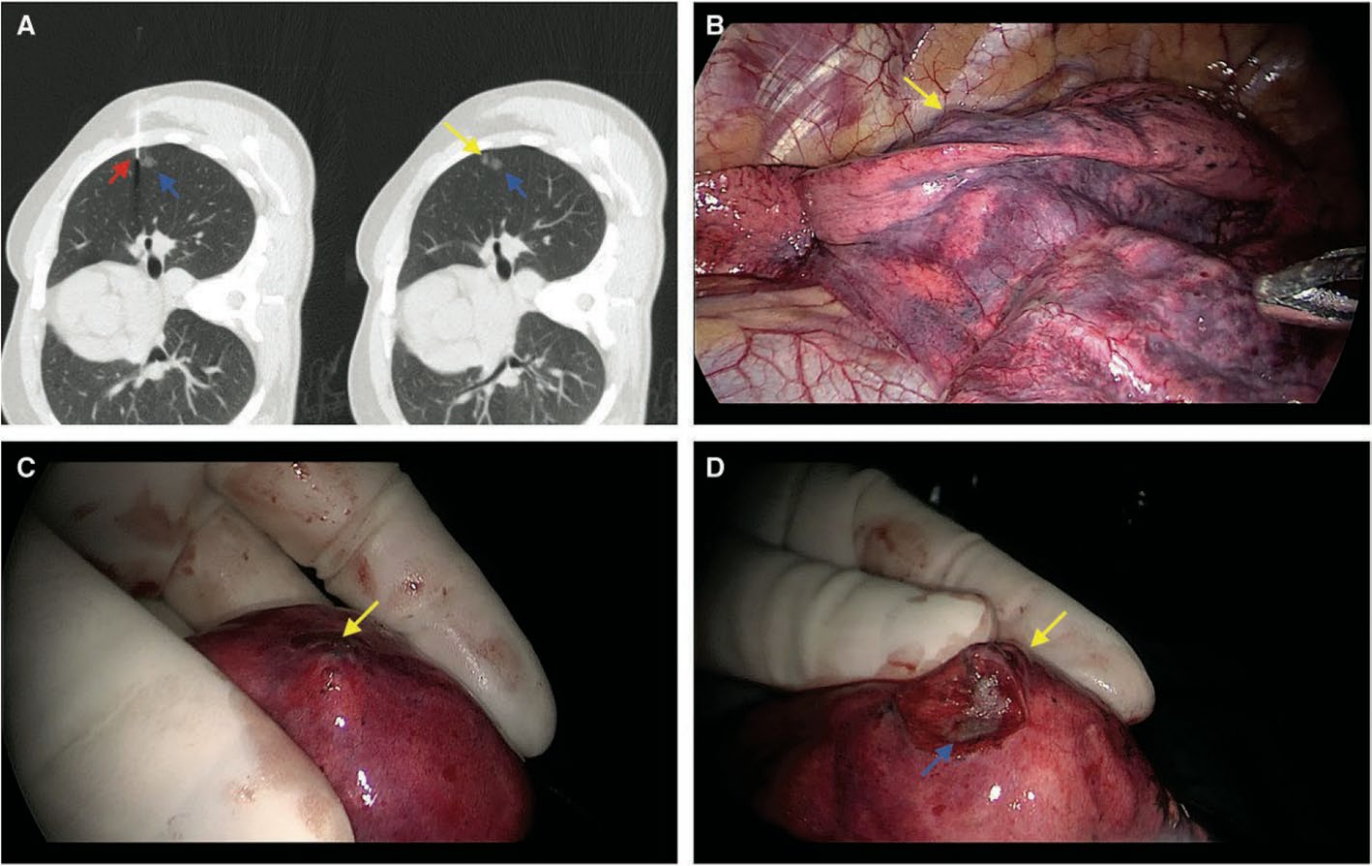
The CT-guided cyanoacrylate localization [6]

The primary component of medical bio-glue is N-butyl-2-cyanoacrylate, known for its non-toxicity and good biological safety. Upon contact with body fluids, it rapidly polymerizes to form a palpable nodule. CT-guided percutaneous injection of bio-glue allows rapid formation within lung tissue, ensuring accurate intraoperative localization. At the same time, the bioglue can persist for several weeks, which means the low requirement for surgical interval. In addition, the rapid coagulation and contraction of bioglue can reduce the risk of pneumothorax and embolism. The disadvantage is that the bioglue has a pungent smell, which can cause obvious irritating cough in patients after entering the bronchus; in addition, bioglue coagulation in the injection needle also has a certain proportion [6]. Despite a slight odor and potential needle clogging, bio-glue demonstrates high success rates, minimal adverse reactions, and good clinical applicability [7]. Figure 3 shows the CT-guided cyanoacrylate localization.

### Contrast agents

**Fig. 4:**
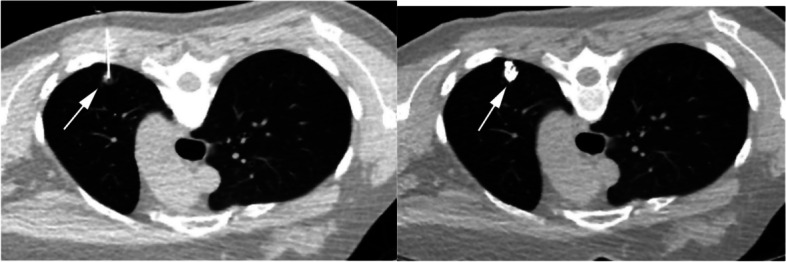
The CT-guided lipiodol localization [10]

Mainly including iodine oil and barium agents, the use of contrast agents implies the need for intraoperative fluoroscopy to confirm localization. Although iodine oil has a longer tissue retention time of three months, its lack of water solubility requires cautious injection to avoid vascular entry and embolic events. Therefore, during iodized oil injection, continuous aspiration and an injection volume of less than 0.5 ml are required [8]. Barium also has a long residual time in the tissue, which is beneficial to surgical planning, but barium may cause mild acute inflammation and edema of the lung parenchyma, which may affect the pathological diagnosis of the target nodule. Therefore, if barium is used, it needs to be injected next to the pulmonary nodule, avoiding direct injection to the target pulmonary nodule [9]. Studies have shown that CT-guided contrast agent localization of pulmonary nodules is simple and efficient [8, 9]. Figure 4 shows the CT-guide lipiodol localization.

### Radioactive elements

The radioactive element used in CT-guided percutaneous pulmonary nodule localization is 99mTc, with a half-life of 6 hours. Gamma probes are employed for intraoperative detection. While operationally straightforward and efficient, the limited post-localization surgical interval and the need for a specialist in Nuclear Medicine and the radiation protection during surgery somewhat restrict its widespread clinical application [11–13].

### Combined material localization

As previously mentioned, each localization material has its own advantages and disadvantages. To further improve localization success rates, some scholars both domestically and internationally have explored combining various materials under CT guidance. Examples include medical bio-glue combined with iodine contrast agents [14], indocyanine green (ICG) combined with iodine contrast agents [15], hook-wire combined with methylene blue [16], hook-wire combined with iodine oil [17], and 99mTc combined with contrast agents [11, 12]. These studies have shown a certain degree of improvement in localization success rates.

The localization rate is over 95% in various materials by CT-guided percutaneous puncture localization [10]. In addition to CT-guided percutaneous puncture localization, there are augmented reality navigation system, electromagnetic navigation-guided system and 3D printed template assisted percutaneous pulmonary nodule localization [18–20]. Li employed the use of Augmented Reality (AR) navigation system and a specialized lung localization marker known as the lungbrella to accurately position percutaneous pulmonary nodules in 10 patients during surgery. Among these patients, successful localization was achieved in 7 cases, while 3 patients experienced failed localization due to software flaws [18]. This technique significantly streamlines the localization process, requiring only a single preoperative CT scan, and allowing for localization to be performed in a standard operating room. This reduction in the time between localization and surgery also minimizes the risk of displacement or shedding of localization materials. However, it is important to note that patients' preoperative CT position must be maintained consistently throughout the surgical procedure. Despite the promising results, the small sample size of this study highlights the need for further improvements and clinical practices to optimize the localization technique. Po-Kuei Hsu and his colleagues attempted electromagnetic navigation-guided percutaneous pulmonary nodule localization, which demonstrates a certain degree of feasibility [19]. As for 3D printed template assisted percutaneous pulmonary nodule localization, we will elaborate in the following sections.

In summary, currently, preoperative CT-guided percutaneous puncture-assisted localization remains the most commonly used method for pulmonary nodule localization. Its primary drawbacks include the risk of complications such as pneumothorax and bleeding, especially in patients with multiple bullae or pneumothorax. Additionally, certain nodules are challenging to localize, such as those near the lung apex, interlobar fissures, close to the diaphragm, or obscured by the scapula. Therefore, the development and exploration of alternative localization techniques on the basis of CT-guided percutaneous puncture-assisted localization are crucial.

## Preoperative bronchoscopic pulmonary nodule localization

In recent years, the field of respiratory intervention has rapidly developed, especially with the emergence of bronchoscopic navigation systems and cone-beam CT (CBCT). This provides new methods for pulmonary nodule localization. Bronchoscopic pulmonary nodule localization allows the localization of nodules that are difficult to target under CT guidance, minimizes the risk of pneumothorax especially for patients suffering from emphysema or COPD.

### Augmented Fluoroscopy Bronchoscopy (AFB) for pulmonary nodule localization

**Fig. 5:**
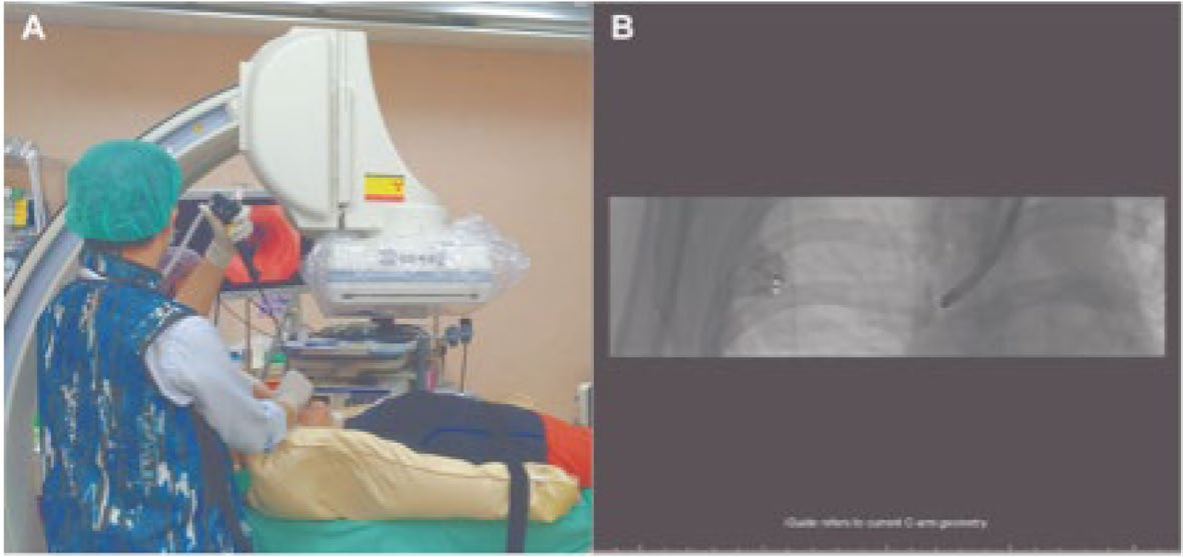
The augmented fluoroscopic bronchoscopic localization [23]

AFB is an advanced bronchoscopy technique that utilizes fluoroscopic imaging to provide real-time visualization of the airways and surrounding structures during bronchoscopic procedures. CBCT employs cone-beam X-rays for a 360-degree scan, providing advantages in three-dimensional reconstruction compared to traditional CT. Under the guidance of CBCT, AFB can provide real-time guidance during bronchoscopic locatlization. Scholars in Taiwan have utilized CBCT navigation-real-time augmented fluoroscopy to inject a mixture of indocyanine green and iodine oil or place micro-spring coils under bronchoscopic guidance [21, 22]. This method involves planning the bronchoscopic route based on CBCT scans, navigating to the target nodule in real-time, and performing injection or placement procedures. The successful localization rate is 94%-100% [21–23]. The mean localization time is (24.1 ± 8.3) min [21]. While demonstrating good safety, high success rates and lower cost than the electromagnetic navigation system, the limitations include the complexity of the procedure, the need for radiation protection, and the potential for multiple path changes during bronchoscopy [23]. Figure 5 shows the augmented fluoroscopic bronchoscopic localization.

### Bronchoscopic navigation system for pulmonary nodule localization

Currently, the widely clinical used bronchoscopic navigation system includes virtual bronchoscopic navigation (VBN), electromagnetic navigational bronchoscopy (ENB) and Augmented reality optical lung navigation (LungPoint navigation technology). VBN, a novel CT-based virtual imaging technology, whose principle involves inputting high-resolution chest CT data into the navigation system, then processing the CT data for path planning, enabling the operator proceeds to the target location through the bronchoscope according to the planned path [24]. Compared to ENB and LungPoint navigation, VBN can’t achieve real-time automatic image matching.

**Fig. 6:**
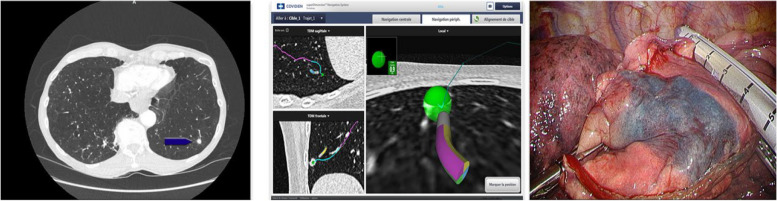
ENB-guided pleural dye marking for a small lung nodule’s localization [25]

ENB is based on electromagnetic localization technology, offering both path planning and real-time localization. The procedure involves inserting a sheath with a navigation line into the bronchoscope's working channel. After matching virtual and real-time images, the sheath is advanced to the target location, allowing for procedures like dye injection or coil placement [25–27]. The reported localization rate is 79%-100%, depending on different localization materials [25–29]. The mean localization time is (12.6 ± 6.5) min [30]. Domestic guidelines recommend the ICG positioning method under ENB [31]. In addition, there are also relevant research reports on the vector positioning method under ENB, which uses the sensor probe of the navigation positioning line as a positioning mark for positioning during VATS surgery; and both the successful resection and localization rates are 100%, the mean localization time is (17.5 ± 4.2) min [32]. Figure 6 shows the ENB-guided pleural dye marking for a small lung nodule’s localization.

**Fig. 7:**
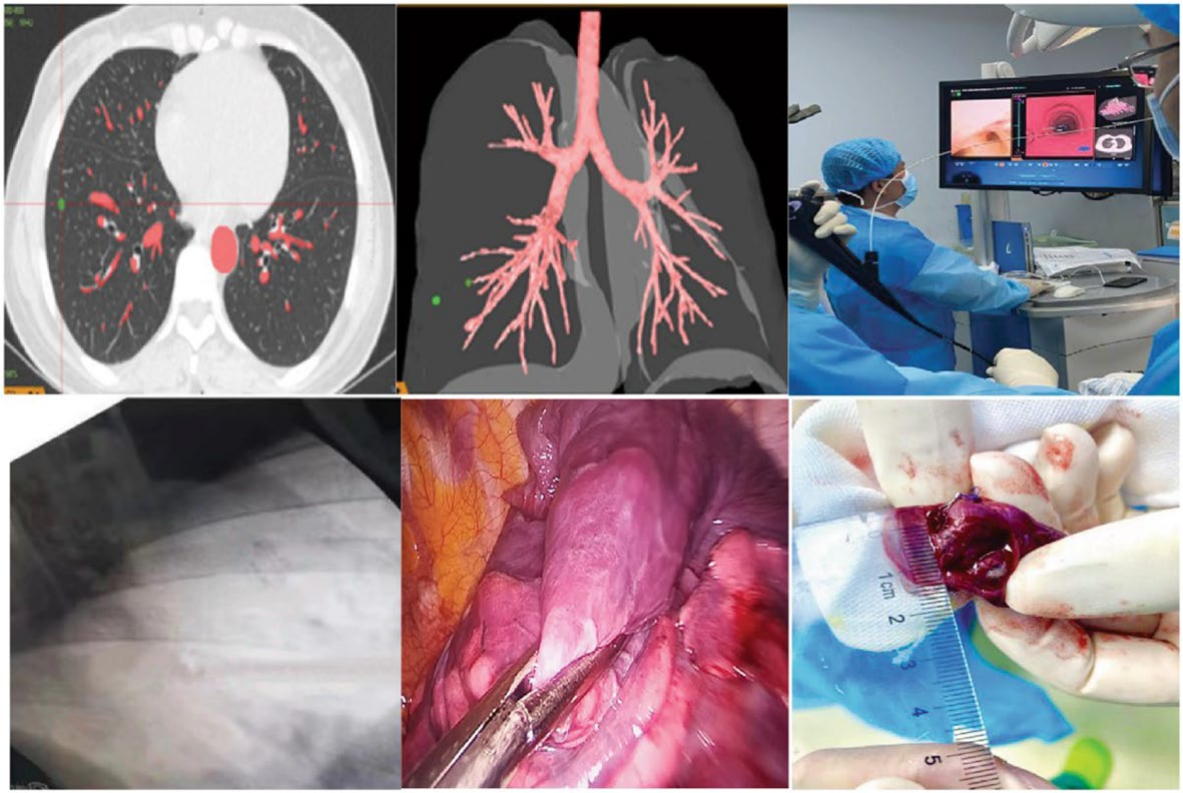
Lungpro-assisted localization with metallic marker [37]

LungPoint is an optical navigation technique that combines augmented reality with real-time matching of endoscopic images based on VBN system. It synchronizes the real bronchoscopic image with the virtual bronchoscopic animation in a dual-channel display format, projecting the planned path onto the real bronchoscopic image in real-time [33]. The augmented reality optical whole lung diagnosis and treatment navigation system (LungPro) is a new generation of optical navigation technology developed by combining infrared optical tracking and navigation technology and fluoroscopy fusion functions based on the previous generation of LungPoint [34]. Yang et al. conducted a comparative study examining the use of CT-guided methylene blue injection localization and Lungpro-assisted ICG injection localization for multiple pulmonary nodules. The results indicated comparable localization efficacy between the two methods [35]. However, for patients with multiple pulmonary nodules, particularly those with bilateral nodules, the CT-guided localization required multiple CT scans and position adjustments, significantly prolonging the localization time. In contrast, Lungpro-assisted localization demonstrated a shorter localization time and lower complication rates. Furthermore, as Lungpro localization is performed under general anesthesia, patients do not experience pain or fear during the procedure, resulting in lower anxiety and depression scores and an enhanced overall patient experience [35, 36]. Figure 7 shows Lungpro-assisted localization with metallic marker.

Both ENB and Lungpoint or lungpro are real-time localization techniques that require the assistance of CBCT and/or endobronchial ultrasound to enhance positioning accuracy. Compared to CT-guided percutaneous localization, navigation localization does not demonstrate a significant advantage in terms of success rate [35], especially for thin and curved target bronchi where bronchoscope and sheath tube insertion is challenging, resulting in potential dissatisfaction with localization. However, the incidence of complications such as bleeding and pneumothorax is significantly reduced [38]. Furthermore, the localization under VBN is faster, especially in patients with multiple nodules, and the patient's experience is improved for the procedure is performed under general anesthesia. However, for ENB localization method, challenges include the high cost and technical expertise required for operators. In addition to the cost of basic equipment, the cost of disposable sheaths and navigation positioning lines is also high. Based on which, ENB has not yet been widely recommended for the localization of pulmonary nodules.

### Virtual-assisted Lung Mapping (VAL-MAP)

**Fig. 8:**
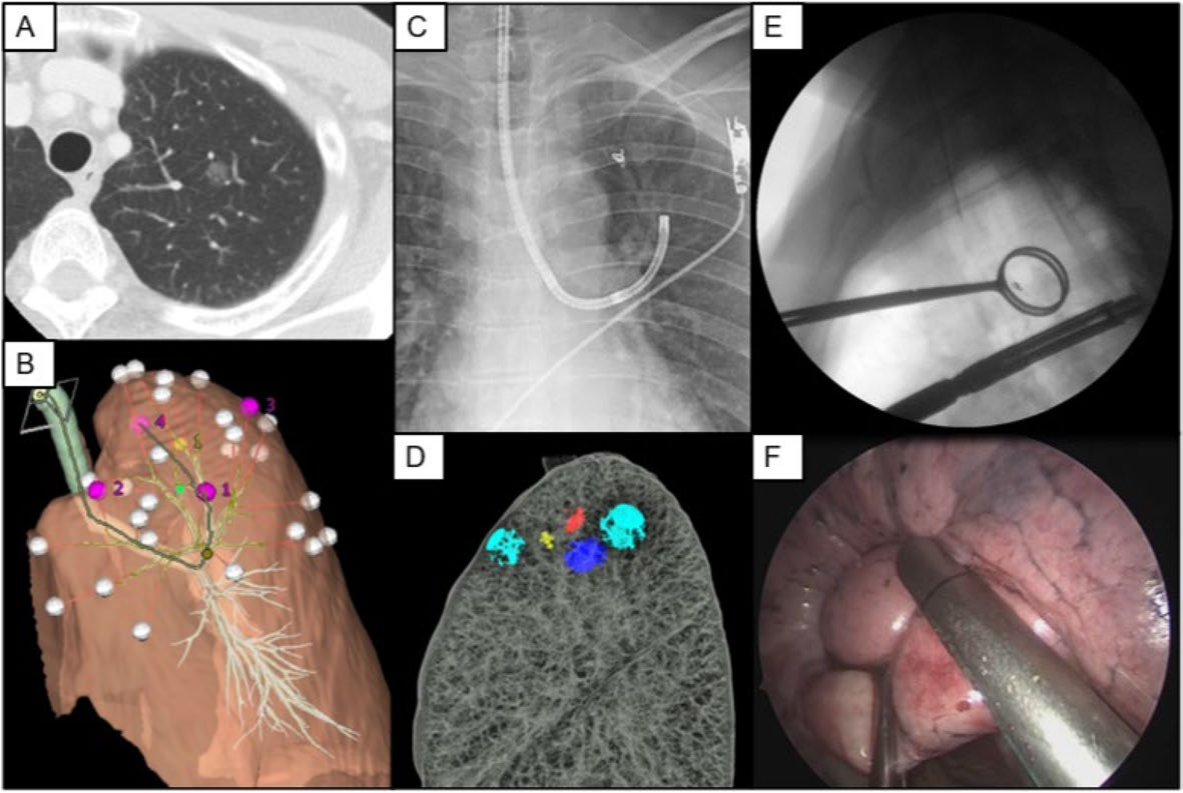
Steps of VAL-MAP 2.0 [43]

VAL-MAP, initially proposed in Japan and widely adopted in their public health system. Surgical team plans the target lesion and surrounding marker points based on preoperative virtual bronchoscopy and 3D imaging. For pulmonary wedge resection, generally 2–3 marker points around the lesion are selected, while for pulmonary subsegmentectomy, 3–4 marker points are chosen. Then, under the guidance of virtual bronchoscopy navigation, indigo carmine is injected for staining. Once the staining is completed, CT scanning and 3D reconstruction are performed again. The resulting 3D reconstruction, also known as the virtual lung map, includes the target lesion and surrounding stained marker points. The surgical margin is adjusted and determined based on the positional relationship between the target lesion and marker points on the VAL-MAP. VAL-MAP is popular in Japan, with VAL-MAP 1.0 being the earliest version used. However, due to its two-dimensional marking technique, information is limited to the pleural surface, making it difficult to obtain adequate resection margins, especially for deeper pulmonary nodules. The emergence of VAL-MAP 2.0 addresses the limitations of version 1.0 by combining multiple staining with microcoil localization for three-dimensional mapping, further precision in resection depth, and improved localization success rates. A multicenter prospective single-arm study showed that successful resection rate was 98.5% under the guidance of VAL-MAP 2.0 [39]. The advantages of VAL-MAP include better determination of surgical margins through multiple localization points, high safety, and effectiveness. However, the process can be cumbersome, costly, and surgery must be performed as soon as possible after localization in case of the diffusion of ICG and displacement of microcoil [39–43]. Figure 8 shows the steps of VAL-MAP 2.0.

### Radiofrequency localization method

**Fig. 9:**
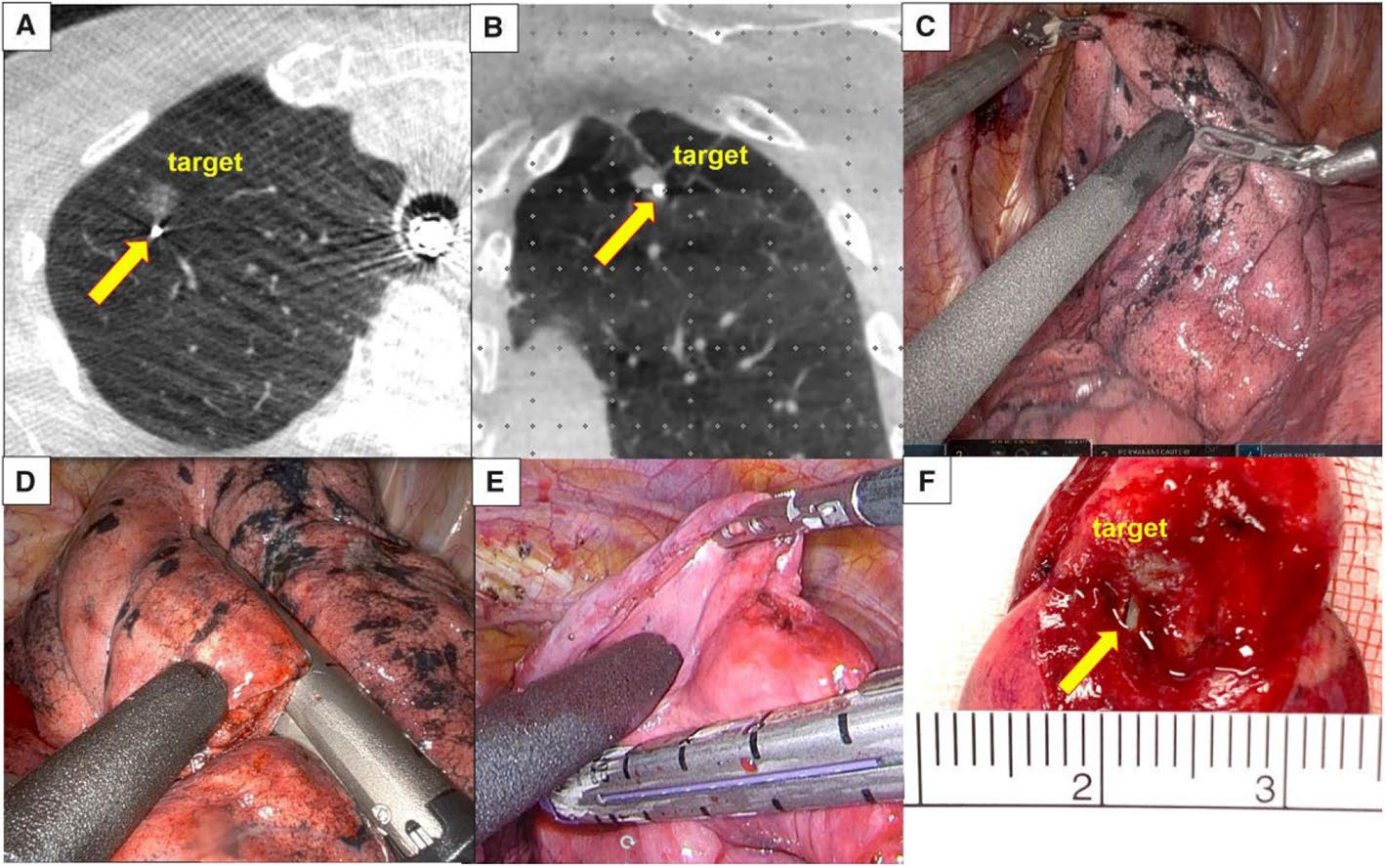
The steps of Radiofrequency identification (RFID) marker placement for wedge resection [47]

Radiofrequency localization method was also first proposed by Japanese scholars and was first applied in humans in September 2019 [44]. The radiofrequency localization method involves placing a radiofrequency identification (RFID) microchip in the targeted bronchi under the guidance of bronchoscopy, VBN or ENB. After confirming the location under CT, use a special probe to detect and locate the microchip in real time during VATS surgery for precise resection. So Miyahara et al. demonstrated the utility and safety of this localization method. The successful localization rate is 98.3%. The average time of placement of RFID microchip was 28.9 ± 15.7 min, and the interval between the placement and surgery can up to 72 hours [45]. Challenges include the need to place the microchip in bronchi smaller than 2mm in diameter to avoid displacement, as well as considerations related to cost and anatomical constraints [45, 46]. Figure 9 The steps of Radiofrequency identification (RFID) marker placement for wedge resection.

### 3D printing-assisted localization

Three-dimensional (3D) printing technology was first described in 1981. Since then, this technology has been widely used in various fields. In the medical field, 3D printing technology shows tremendous potential in preoperative planning, intraoperative guidance, and medical education.

It is reported that there are three methods for pulmonary nodule localization by the 3D Printing-assisted location system, among which the most common one is the use of 3D printed templates navigation to assist percutaneous puncture localization. 3D printed template navigation utilizes 3D printing technology to create a percutaneous puncture localization plate, which is attached to the patient's chest wall before surgery, allows for the guidance of needle insertion along the predefined needle path to locate pulmonary nodules [20, 48, 49]. Compared to CT-guided puncture localization, the advantage of this localization method is to simplify the puncture localization procedure and reduce radiation dose, potentially allow for percutaneous localization without relying on CT scans. The mean localization time is 9.8 ± 2.0 min [48]. Zhang et al. compared the hookwire placement methods guided by CT and 3D-printed template navigation, finding that the latter is not inferior to the former in terms of effectiveness, and is safer and simpler [20]. Rui Fu's study also showed that the 3D-printed template navigation localization has a good effect, especially for accurate localization of nodules in the upper lobe of the right lung. However, this technology is affected by respiration and body position, and patients with excessively high BMI may compromise the template fit, thereby affecting the positioning accuracy [50, 51]. Another type of 3D-printed template-assisted localization is noninvasive. The chest high‐resolution CT image data were utilized for digital reconstruction and 3D printing to make a tailored life‐size emulation pulmonary nodules localization model, which was placed beside the thoracoscope screen. The surgeon refers to the model and performs intraoperative positioning according to the map by utilizing the spatial geometric relationship such as the distance and proportion between various anatomical landmarks of the lungs and pulmonary nodules [52]. The third 3D printing localization method is reported by Tang et al. in their study of 12 patients undergoing localization, they printed a flexible 3D material as an intraoperative guide, then placed the 3D model inside the chest cavity during surgery for guidance [53]. Although the literature reports that the accuracy of the latter two localization methods is as high as 100% [52, 53], due to the small sample size, the accuracy and efficiency of localization need further verification.

**Fig. 10:**
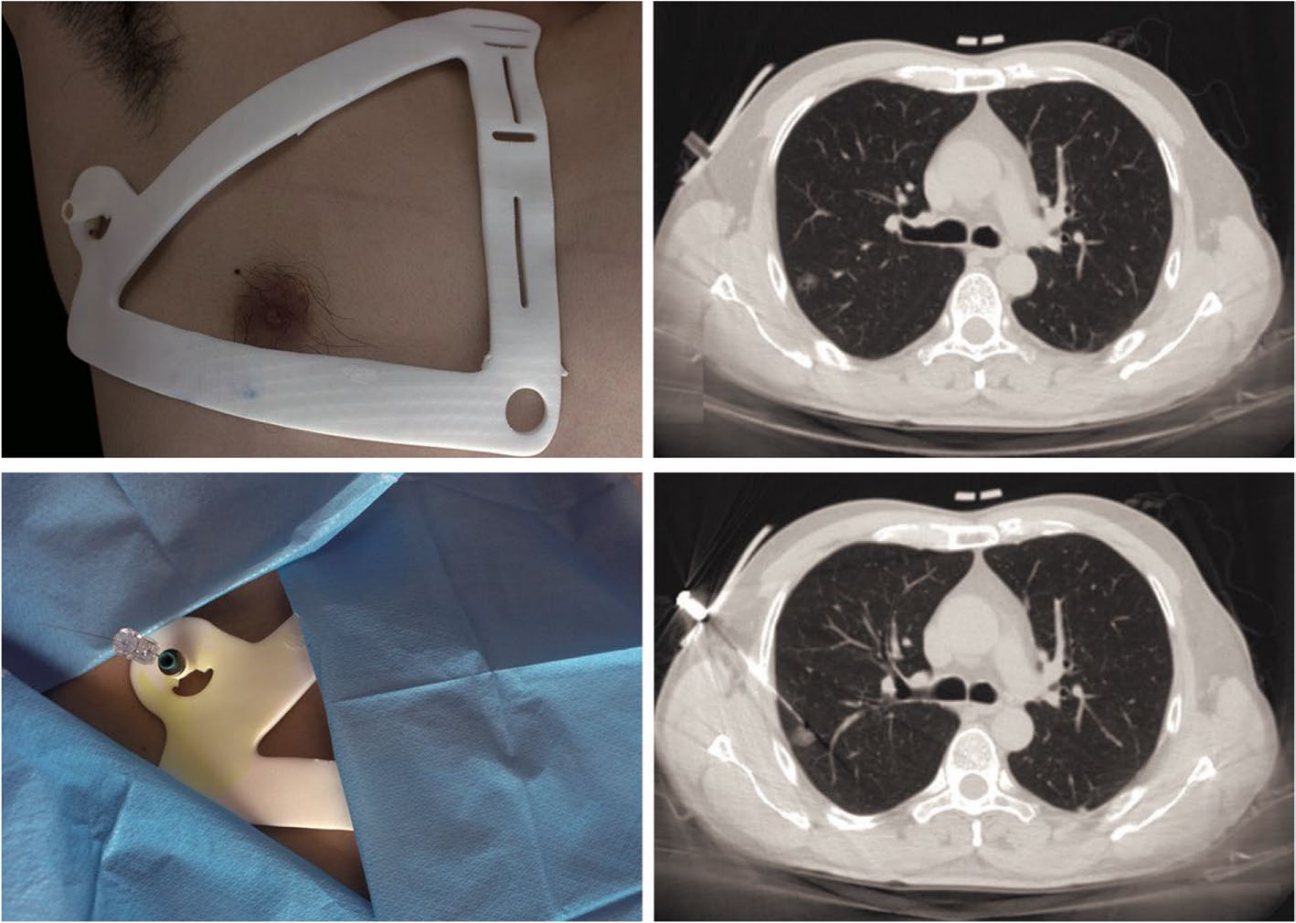
3D printed templates navigation to assist percutaneous puncture localization [20]

In general, 3D printing technology provides a more intuitive, accurate, and efficient tool for the localization of pulmonary nodules, which helps to improve the precision and efficiency of surgery and reduce radiation exposure. It has great development potential in the future. Figure 10 shows 3D printed templates navigation to assist percutaneous puncture localization.

## Intraoperative pulmonary nodule localization under ultrasound guidance

**Fig. 11:**
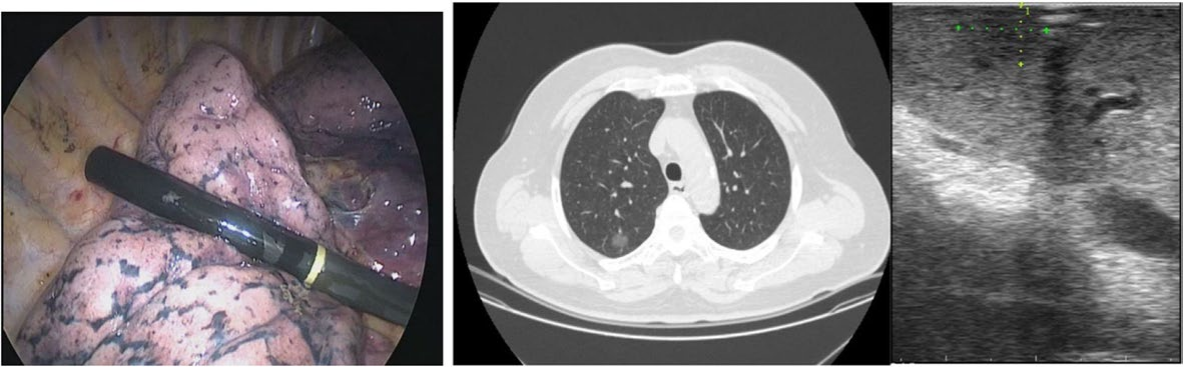
Intraoperative ultrasound localization of pulmonary nodules [54]

Intraoperative ultrasound-guided pulmonary nodule localization refers to the use of ultrasound to locate and resect nodules during single-lung ventilation. During VATS surgery, the ultrasound probe can explore almost the entire visceral pleura and detect mediastinal nodules for staging assistance. However, the quality of ultrasound imaging may be affected by residual air in the lungs, which is why most studies exclude patients with COPD, emphysema, and asthma. Research suggests that the accuracy rate of intraoperative ultrasound localization is over 90%, and the positive localization rate for mixed density or solid nodules is higher than that of GGO patients [47, 54]. Additionally, intraoperative ultrasound can serve as a compensatory localization method when CT-guided intraoperative localization fails. Overall, intraoperative ultrasound (ILU) is a real-time, safe, and economical localization method. During the procedure, lung collapse is essential for accurate localization, and an experienced ultrasound doctor is required, especially for patients with GGO and multiple emphysema. Figure 11 shows the intraoperative ultrasound localization of pulmonary nodules.

As shown in Table 1, each localization method has its advantages and disadvantages. Apart from the success rate of localization, more comprehensive consideration should be given to various factors when selecting the localization method for pulmonary nodules, including the location and size of the lung nodules, the distance from the pleural membrane, cost, individual patient conditions, equipment such as CT, ENB, and ultrasound, as well as the availability of corresponding professionals. If the patient is generally in good condition, without multiple emphysema, and the lung nodule is not located near the lung apex, interlobar fissures, close to the diaphragm, or obscured by the scapula, we prefer CT-guided percutaneous lung nodule localization. Metal localization materials are our first choice. If the hospital is equipped with a fluorescent thoracoscope, ICG can be selected, too. For patients unsuitable for percutaneous lung puncture localization due to conditions such as multiple emphysema, the special nodule location, or patient's posture difficulties, the option of bronchoscopic localization under general anesthesia can be chosen. ICG is recommended as the localization material. For hospital equipped with VBN and the nodule location is easily navigable, VBN is prioritized. For nodule which is located in the distal bronchi and requires real-time localization, ENB or Lungpro-assisted localization can be selected. For hospital without navigation bronchoscope, intraoperative ultrasound localization can be considered, but it should be entrusted to an experienced ultrasonographer. VAL-MAP needs to be performed under ENB and is rarely used in domestic hospitals. For patients with pulmonary nodules that are difficult to identify under thoracoscopy or those who have difficulty determining the resection margin during sublobar resection, VAL-MAP-assisted localization can be considered. 3D printing-assisted localization and radiofrequency localization of pulmonary nodules have not been widely used in hospitals, and a large amount of clinical data is needed to verify their clinical practicality. Therefore, detailed indications are not recommended here temporarily. We believe that with the development of science and technology, new technologies like 3D-Printing, VAL-MAP will provide better services for lung nodule localization.

**Table 1:** Summary of commonly used pulmonary nodule localization methods

Localization method	Instrument	Localization materials	Advantage	Disadvantage
Percutaneous puncture-assisted localization	CT; augmented reality navigation system; electromagnetic navigation system; 3D printing technology	Metallic Materials; Dyes; Medical bio-glue; Contrast agents; Radioactive elements	- Easy to operate, short operation time and widely used - A variety of selectable localization materials - High success and low cost	- Pneumothorax; hemorrhage - Difficult to localize nodules in certain locations - Need to consider surgical interval
Bronchoscopic pulmonary nodule localization	AFB	Indocyanine green; iodine oil; Metallic Materials	- Safety - High success rates and lower cost	- Complexity of the procedure - Need for radiation protection - Possible multiple path changes during bronchoscopy
	ENB	Dyes; Metallic Materials; Sensor probe	- Real-time localization, improving localization accuracy - Safety	- High equipment costs - Requires specialized operators
	VAL-MAP	Indigo carmin; Metallic Materials;	- Better determination of surgical margins - High safety and effectiveness	- Cumbersome process and high costs - Limited surgical interval
	Radiofrequency	Microchip	- Real-time detection, precise resection - Safety	- High cost - Anatomical constraints for the localization of microchip
3D printing-assisted localization	3D-printed template	Metallic Materials; Methylene blue; 3D model; or none	- Improve surgical precision and efficiency - Reduces radiation exposure - The accurate localization of nodules in the upper lobe of the right lung	- Affected by respiration and body position - High BMI patients may affect localization accuracy - Additional printing costs and time
Intraoperative ultrasound localization	Ultrasound	None	- Real-time, safe, and economical localization method - Serve as a compensatory localization method	- Ultrasound imaging quality may be affected by residual air in the lungs - Requires experienced ultrasound doctors

## Data Availability

The datasets generated and analyzed during the current study are not publicly available but are available from the corresponding author on reasonable request.
